# Epidemiology of Traumatic Injuries at a Single Regional Trauma Center in South Korea: Age-Specific and Temporal Trends (2014–2023)

**DOI:** 10.3390/healthcare13070773

**Published:** 2025-03-31

**Authors:** Sebeom Jeon, Gil-Jae Lee, Mina Lee, Kang-Kook Choi, Seung-Hwan Lee, Sung-Youl Hyun, Yang-Bin Jeon, Byungchul Yu

**Affiliations:** 1 Department of Trauma Surgery, Gachon University Gil Medical Center, Incheon 21565, Republic of Korea; dsjeonse@gmail.com (S.J.); nonajugi@gilhospital.com (G.-J.L.); jwh@gilhospital.com (M.L.); choikangkook@gilhospital.com (K.-K.C.); surgeonrumi@gmail.com (S.-H.L.); sungyoul@gilhospital.com (S.-Y.H.); junyb@gilhospital.com (Y.-B.J.); 2 Department of Traumatology, Gachon University College of Medicine, Incheon 21565, Republic of Korea

**Keywords:** trauma centers, regional trauma systems, epidemiology, injury severity score, retrospective studies, wounds, injuries

## Abstract

**Background/Objectives**: Trauma remains a leading cause of morbidity and mortality worldwide, significantly affecting younger and aging populations. The regional trauma center establishment in South Korea in 2014 marked a pivotal advancement in the national trauma care system. This study reviews data from the Incheon Regional Trauma Center, analyzing patient demographics, injury mechanisms, and outcome trends. **Methods**: This retrospective analysis included 32,025 patients with trauma treated at the Incheon Regional Trauma Center from 2014 to 2023. Data from the Korean Trauma Database included demographics, injury mechanisms, Injury Severity Scores (ISSs), and outcomes. We considered the initial treatment setting in our analysis to evaluate its potential impact on patient outcomes. Patients were stratified into four ISS categories and five age groups (0–12, 13–19, 20–64, 65–79, and ≥80 years). Statistical comparisons used chi-square tests and analysis of variance. **Results**: The proportion of older patients (≥65 years) increased significantly from 23.3% in 2014 to 45.2% in 2023, with patients aged ≥ 80 years showing the highest rise and mortality (6.7%). Traffic accidents decreased from 30.7% to 28.2%, while slip injuries increased from 26.9% to 34.0%. Patients with ISSs > 15 increased, peaking in 2022 (mortality, 15.6%). Despite improved trauma care infrastructure, the overall mortality rate reached 5.9% in 2022, possibly due to more complex cases and an aging population. **Conclusions**: This study highlights evolving challenges in trauma care due to demographic shifts and rising injury severity. These findings underscore the need for tailored geriatric trauma care, improved infrastructure, and targeted interventions for high-risk groups. Further research is needed to optimize trauma systems and address the growing burden of geriatric trauma.

## 1. Introduction

Trauma remains a major global health concern and one of the leading causes of mortality worldwide [1,2,3]. Approximately 4.4 million people die annually due to traumatic injuries, accounting for approximately 8% of all deaths [4]. Among individuals aged < 35, trauma is the predominant cause of death and disability, highlighting trauma’s critical impact on younger populations [5]. These statistics emphasize the urgent need for effective trauma care systems to reduce mortality and improve outcomes for trauma patients.

In South Korea, the introduction of regional trauma centers in 2014 marked a significant milestone in the development of a national trauma care system. Before their implementation, the preventable trauma mortality rate was high (35.2%), as reported in 2012 [6,7]. This high mortality rate underscored the pressing need for an organized and efficient trauma care system. The year 2014 was a pivotal moment, as it marked the establishment of a nationwide strategy to address these deficiencies and improve trauma care outcomes [8].

The Incheon Regional Trauma Center is one of the first trauma centers in South Korea and was established in 2014 [9,10]. Over the past decade, it has played a critical role in the management of patients with trauma. Incheon, with approximately 3 million residents, is the third most populous city in the country. The Incheon Regional Trauma Center serves not only Incheon but also surrounding areas, providing trauma care services to an estimated population of 7 million. This extensive catchment area underscores the center’s importance in the South Korean trauma care network.

This study aimed to examine the demographic characteristics, injury patterns, and initial treatment settings of patients with trauma treated at the Incheon Regional Trauma Center over a 10-year period (2014–2023). By analyzing the data collected during this time, we aim to provide a comprehensive overview of trauma care trends and changes in South Korea. This report presents the findings of a decade of data collection, highlighting the contributions of the Incheon Regional Trauma Center to improve trauma outcomes and offering valuable insights for further advancements in trauma care systems.

## 2. Materials and Methods

### 2.1. Study Population

This study retrospectively analyzed 32,025 trauma patients who presented to our regional trauma center over a 10-year period. This study included trauma patients who presented to the Incheon Regional Trauma Center between 1 January 2014 and 31 December 2023 and were registered in the Korean Trauma Database (KTDB). Cases with incomplete data that precluded analysis were excluded, as well as patients who died prior to hospital arrival following injury. In South Korea, regional trauma centers are designated as the highest level of trauma care, comparable to Level 1 trauma centers in other systems. These centers are critical for managing severe trauma cases nationwide by providing comprehensive, multidisciplinary care.

### 2.2. Data Source

Data for this study were obtained from the KTDB, a nationwide trauma registry that collects prospectively recorded data from trauma centers across South Korea. The KTDB is a standardized database designed to monitor and enhance the quality of trauma care. To ensure data accuracy and completeness, the center conducts regular trauma team meetings with a multidisciplinary group comprising trauma surgeons, supporting specialists, trauma coordinators, and staff from various fields. The dataset includes patient demographics, pre-hospital information (e.g., mode of transportation and pre-hospital time intervals), mechanisms of injury, and hospital data, such as ED arrival times, initial vital signs, and transfusion volumes. Additional variables include Injury Severity Score (ISS), admission status, length of hospital stay, and discharge outcomes. These comprehensive data points enable an in-depth analysis of injury patterns and outcomes in real-world trauma care settings.

### 2.3. Patient Stratification and Injury Classification

For detailed analysis, patients were stratified into five age groups: 0–12 years, 13–19 years, 20–64 years, and ≥65 years. Injury mechanisms were classified into major categories, including traffic accidents, falls, slips, stab injuries, being struck by objects, and others. Traffic accidents encompassed injuries from motor vehicle, motorcycle, bicycle, and pedestrian accidents. The ‘others’ category included gunshot wounds, drowning, asphyxiation, burns, and cases with unknown mechanisms. Only one gunshot wound was reported in the past decade. Patients were also classified by their initial treatment setting, distinguishing between those admitted directly to the trauma bay and those who arrived via the general emergency room (ER). Injury severity was analyzed by grouping patients into four categories based on ISS: 1–8, 9–15, 16–24, and ≥25. Mortality was analyzed in two categories: deaths occurring at the trauma bay stage and those after hospital admission. Trauma bay deaths included patients declared dead on arrival (DOA), those arriving while undergoing cardiopulmonary resuscitation (CPR) but not resuscitated, and those who underwent CPR initiated in the trauma bay but did not survive.

### 2.4. Statistical Analysis

Differences between groups were assessed using the chi-square test for categorical variables, such as injury mechanisms and patient outcomes. The normality of continuous variables was assessed using the Kolmogorov–Smirnov test, and the homogeneity of variances was examined using Levene’s test. Continuous variables, including patient age, time intervals, and Injury Severity Scores, were analyzed using one-way analysis of variance (ANOVA) for comparisons across multiple groups. Following the one-way ANOVA, we conducted a post-hoc analysis to determine which groups differed significantly. Appropriate multiple comparison procedures were employed based on the assumption of homogeneity of variances, ensuring accurate detection of group-level effects. Categorical variables are presented as frequencies and percentages, while continuous variables are reported as mean ± standard deviation (SD) or median with interquartile range (IQR), depending on the data distribution. *p*-values < 0.05 were considered statistically significant. All statistical analyses were performed using SPSS (version 22.0 for Windows; SPSS Inc., Chicago, IL, USA). This study adhered to ethical standards and was approved by the Institutional Review Board (IRB) of our institution (approval number: GBIRB2025-009). Patient data were anonymized to ensure confidentiality, and all analyses followed relevant guidelines and regulations.

## 3. Results

### 3.1. Patient Demographics

The baseline characteristics of 32,025 trauma patients reveal that 62.9% were male, with a mean age of 52.8 ± 22.2 years. Traffic accidents (29.9%, n = 9019), slips (26.9%, n = 8134), and falls (19.6%, n = 5923) were the leading mechanisms of injury, accounting for more than three-quarters of all cases. Less common mechanisms included being struck by objects (11.2%, n = 3378) and penetrating injuries (6.9%, n = 2084). The overall median ISS was nine (IQR 4–13), and 21.4% sustained severe injuries (ISS > 15, n = 6168). Among injuries with an Abbreviated Injury Scale (AIS) of three or higher, head injuries had the highest incidence at 18.1%. Torso injuries of AIS three or higher, including both thoracic and abdominal regions, accounted for 20%. At initial triage, 33.4% (n = 10,711) were categorized as trauma bay cases. A total of 70.2% (n = 22,358) arrived directly at the hospital, while 29.8% (n = 9474) were transferred from other hospitals. The mean hospital length of stay (HLOS) was 13.9 ± 16.8 days. Overall, 53.6% were discharged home, while 40% were required to transfer to rehabilitation facilities, long-term care facilities, or other hospitals. Among the total population, 2.4% (n = 774) were declared dead on arrival (DOA), including those who died after cardiopulmonary resuscitation (CPR) in the emergency room. Additionally, 3.9% (n = 1256) expired after admission (Table 1).

### 3.2. Yearly Trends

**Figure 1:**
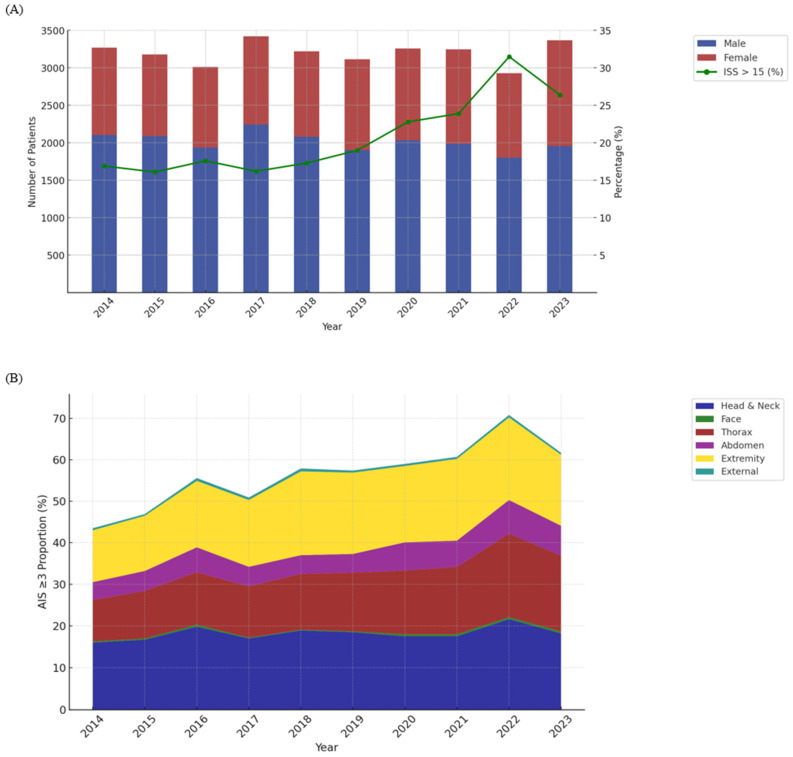
Annual number of patients and yearly trend of proportion of patients with an Injury Severity Score > 15 (**A**) and trend of AIS ≥ 3 proportion by body region (2014–2023) (**B**).

Figure 1A shows the annual number of trauma patients (blue bars) and the yearly trend in the proportion of patients with an ISS > 15 (green line) during the study period from 2014 to 2023. Patient numbers remained >3000 yearly, except for 2022, when it dropped to 2930. The proportion of severely injured patients with an ISS > 15 ranged between 16.0% and 19.0% until 2019, exceeded 20% for the first time in 2020 (22.8%), and peaked in 2022 (>31.5%) (Table S1). From 2014 to 2023, the overall proportion of patients with AIS ≥ three injuries increased across all body regions (Figure 1B). Notably, significant increases were observed in thoracic (9.9% to 18.2%) and extremity injuries (12.5% to 17.1%), while head and neck injuries remained consistently high, ranging from 16.0% to 21.6%. Abdominal injuries also increased modestly, from 4.3% to 7.2% (Table S2).

**Figure 2:**
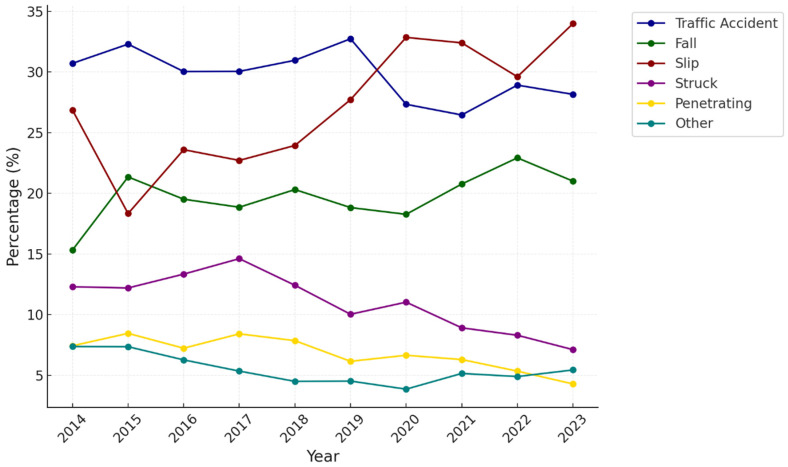
Trend in the proportion of patients by injury mechanisms.

The distribution of trauma mechanisms from 2014 to 2023 reveals distinct trends (Figure 2). Traffic accidents decreased overall, dropping from 30.7% (n = 1004) in 2014 to 28.2% (n = 807) in 2023. This decline was consistent throughout the study period, with the lowest proportion in 2022 (26.5%, n = 714). Slip injuries steadily increased from 26.9% (n = 878) in 2014 to 34.0% (n = 974) in 2023, peaking in 2023. This upward trend was particularly notable between 2018 and 2023 when slips consistently accounted for more than 29% of cases yearly. Falls slightly increased, rising from 15.3% (n = 501) in 2014 to 21.0% (n = 602) in 2023. Injuries caused by being struck by objects decreased throughout the study period, declining from 12.3% (n = 402) in 2014 to 7.7% (n = 204) in 2023. This reduction was consistent, with the largest decline observed between 2017 and 2023. Similarly, penetrating injuries steadily decreased from 7.4% (n = 243) in 2014 to 4.3% (n = 123) in 2023 (Table S3).

**Figure 3:**
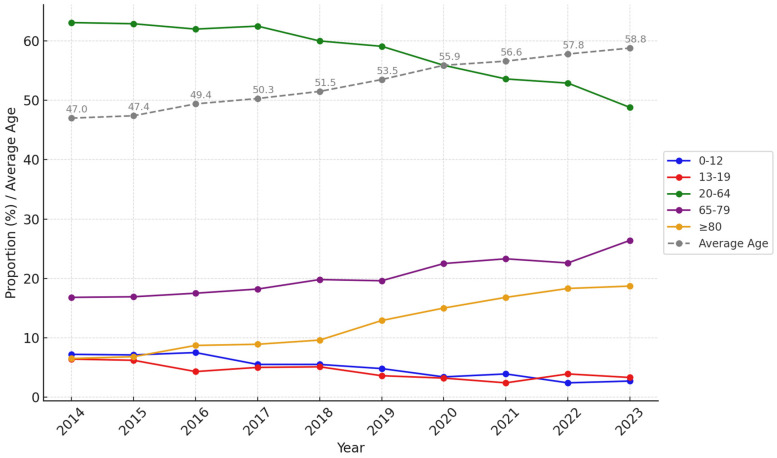
Trends in age group proportions and average age (2014–2023).

From 2014 to 2023, the proportions of elderly (65–79 years) and super-elderly (≥80 years) patients with trauma steadily increased. The average age of patients with trauma also consistently rose by 11.8%, from 47.0 years in 2014 to 58.8 years in 2023 (Figure 3). In 2023, patients aged ≥ 65 years accounted for 45.2% (n = 1523) of all trauma cases, nearly half of the total cases. The proportion of super-elderly patients increased from 6.5% (n = 212) in 2014 to 18.7% (n = 632) in 2023, a rise of 12.2%, higher than the 9.6% increase in elderly patients aged 65–79 years over the same period (Table S4).

**Figure 4:**
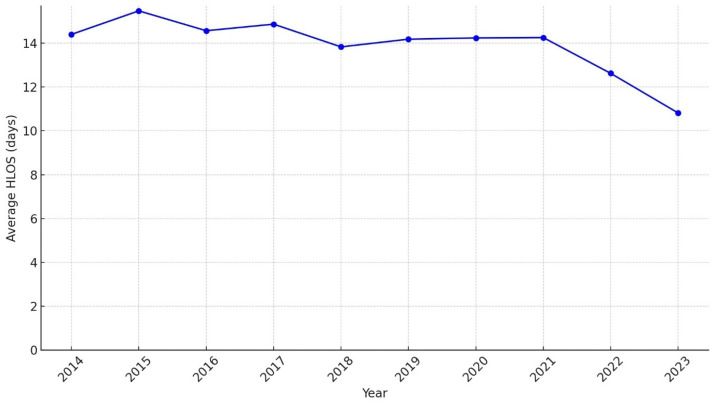
Trend of hospital length of stay for all patients with trauma (2014–2023).

The annual trend in HLOS for patients with trauma revealed a gradual decline over time (Figure 4). The average HLOS was 14.41 days in 2014 and peaked slightly at 15.47 days in 2015. However, from 2016 onward, the average HLOS showed a steady downward trend, reaching 10.82 days in 2023, a reduction of approximately 5 days compared to 2015.

### 3.3. Age Group

Table 2 summarizes the demographic and clinical characteristics of patients with trauma categorized by age groups (0–12, 13–19, 20–64, 65–79, and ≥80 years). The proportion of male patients decreased with age, from 73.7% (n = 1024) in the 13–19 group to 71.9% (n = 13,369) in the 20–64 group, 52.3% (n = 3417) in the 65–79 group, and 33.1% (n = 1291) in the ≥80 group.

Injury mechanisms varied significantly across age groups. Falls were most common in the 0–12 group (30.8%, n = 483). Traffic accidents were predominant in the 20–64 group (33.1%, n = 5905). Among the 65–79 group, slips were most common (40.4%, n = 2418), followed by traffic accidents (27.9%, n = 1672) and falls (18.4%, n = 1102). Similarly, in the ≥80 group, slips accounted for 63.6% (n = 2189), followed by traffic accidents (13.9%, n = 479) and falls (15.1%, n = 520). Injury severity also differed across age groups. The median ISS was lowest in the 0–12 group [4 (IQR 2–5)], higher in the 20–64 group [6 (IQR 4–14)], and increased with age in the 65–79 group [9 (IQR 4–14)] and ≥80 group [9 (IQR 5–10)]. The proportion of patients with severe injuries (ISS > 15) was 7.9% (n = 125) in the 0–12 group, 17.5% (n = 238) in the 13–19 group, 22.0% (n = 3936) in the 20–64 group, 22.6% (n = 1349) in the 65–79 group, and 15.2% (n = 520) in the ≥80 group.

HLOS increased with age, with an average of 5.3 ± 8.9 days in the 0–12 group, 11.2 ± 15.2 days in the 13–19 group, 14.3 ± 18.0 days in the 20–64 group, 15.2 ± 16.3 days in the 65–79 group, and 14.6 ± 13.9 days in the ≥80 group. Discharge disposition also varied by age, with 74.3% (n = 2220) of patients aged 19 and under being discharged home, compared to 54.9% (n = 10,757) of adult patients, 48.9% (n = 3179) of patients aged 65–79, and 42.9% (n = 1670) of patients aged ≥80.

### 3.4. Mortality

**Figure 5:**
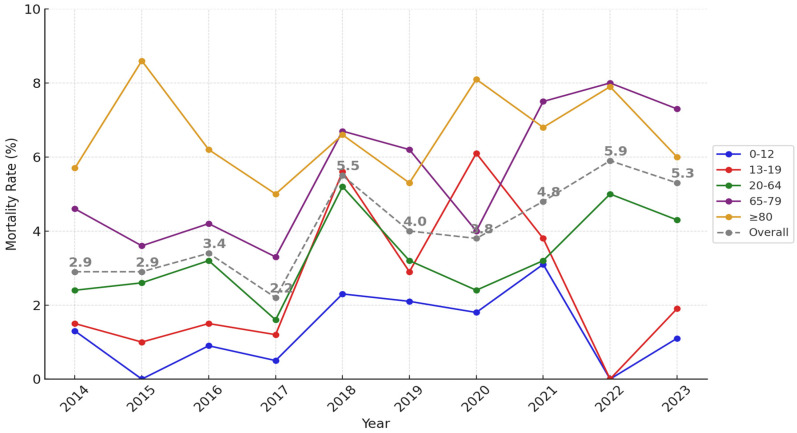
Trends in mortality rates by age group and year (2014−2023).

The yearly trend in mortality from 2014 to 2023 shows an overall increase over the study period despite some fluctuations (Figure 5). Overall mortality remained stable at 2.9% in 2014 and 2015 but dropped to a low of 2.2% in 2017. From 2018, a steady upward trend was observed, peaking at 5.9% in 2022 before decreasing slightly to 5.3% in 2023. Age group-specific trends revealed distinct patterns. In the 0–12 age group, mortality remained consistently low at 1.3% throughout the study. For the 20–64 age group, the highest mortality rate was 5.2% in 2018, with an overall mortality rate of 3.2%. Among patients aged 65–79, mortality remained < 5%, with a low of 3.3% in 2017, but increased significantly to 6.6% in 2018 and further to 8.0% in 2022, showing a clear upward trend. Among the super-elderly patients (≥80 years), mortality rates consistently exceeded 5% throughout the entire period, with an overall average mortality rate of 6.7% (Table S5).

Mortality differed significantly by age group, mechanism of injury, and injury severity (Table 3). Among age groups, super-elderly patients (≥80 years) had the highest mortality (6.7%), followed by elderly patients (65–79 years) at 5.7%. Adults (20–64 years) had a mortality of 3.2%, while adolescents (13–19 years) had a mortality of 2.4%. Children (0–12 years) had the lowest mortality at 1.3%. Regarding mechanisms of injury, falls were associated with the highest mortality (5.5%), followed by traffic accidents (4.7%) and slips (3.2%). Patients with severe injuries (ISS ≥ 25) exhibited the highest mortality at 26.4%, compared to 6.9% for those with an ISS of 16–24, 1.8% for those with an ISS of 9–15, and just 0.5% for those with an ISS of 1–8 (Table S6).

## 4. Discussion

This study provides valuable insights into the evolving epidemiology and outcomes of traumatic injuries by analyzing patients with trauma treated at a regional trauma center in South Korea over a 10-year period. The findings highlight the need for strategic adjustments in trauma care to address changes in demographics, injury mechanisms, and patient outcomes. To our knowledge, this is the first epidemiological analysis over such an extended period to examine the demographics and injury patterns of a South Korean regional trauma center.

The Incheon Regional Trauma Center, established in 2014, is one of the largest trauma centers in South Korea, playing a specialized role in advancing the regional trauma system and providing definitive care for trauma patients. The center operates 24 h a day with dedicated trauma surgeons, anesthesiologists, and other essential personnel, supported by facilities, such as a trauma bay, trauma-dedicated operating rooms, and intensive care units. Over time, our center has steadily expanded its trauma-dedicated workforce and adopted a multidisciplinary approach to care, enabling faster evaluations, more coordinated treatment plans, and ultimately a reduction in hospital length of stay. Additionally, continued investment in trauma-specific infrastructure has helped streamline patient flow and reinforce the center’s capacity to handle increasingly complex cases.

Additionally, the center established a trauma registry based on the KTDB and continuously implemented comprehensive quality assessment, management, and improvement measures, treating >3000 patients with trauma annually, with an average of 616 patients yearly having an ISS > 15. According to the “Resources for Optimal Care of the Injured Patient”, updated by the American College of Surgeons in 1999, a Level 1 trauma center must treat at least 1200 admissions or 240 severe trauma cases annually, with each trauma surgeon managing an average of 35 patients with severe trauma [11,12,13]. Our trauma center’s workforce, facilities, equipment, and quality management meet the criteria for a Level 1 trauma center in the United States.

Notable trends were observed in the distribution of injury mechanisms during the study period. While traffic accidents remained the leading cause of overall trauma, their proportion decreased from 30.7% in 2014 to 28.2% in 2023. A significant turning point in the declining trend of traffic accidents was observed beginning in 2020. This decrease may reflect the impact of reduced speed limits implemented in Incheon, with pilot enforcement starting in October 2019 and full implementation in April 2021. Reduced speed limits have the potential to significantly lower the frequency of traffic accidents and associated fatalities, contributing to improved road safety and the prevention of severe injuries [14,15]. Meanwhile, falls were the most common injury mechanism among children aged < 12 years (30.8%) and the second most common among adolescents (15.1%), potentially linked to increasing suicide attempts in South Korea, which has the highest suicide mortality rate among the Organization for Economic Cooperation and Development (OECD) countries. In 2019, South Korea’s suicide rate was 24.6 per 100,000 people, ranking first among OECD nations [16]. The increasing suicide rate among adolescents is particularly concerning. For adolescents aged 10–14 years, the rate more than doubled, increasing from 0.8 per 100,000 in 2000 to 1.9 per 100,000 in 2019, representing an alarming trend [17]. Addressing the increasing adolescent suicide rate requires continuous and comprehensive efforts, including developing and implementing educational and preventive programs to support vulnerable adolescents [18]. Collaboration among schools, families, healthcare providers, and policymakers is essential for creating a safe and supportive environment for adolescents [19,20].

Despite improvements in trauma care infrastructure, the overall mortality rate peaking at 5.9% in 2022 remains a concerning trend. Several factors may contribute to this gradual increase. First, the rising proportion of elderly patients with trauma (aged ≥ 65 years) within the total trauma population is likely significant. The proportion of elderly patients with trauma steadily increased from 23.3% in 2014 to 45.2% in 2023—a striking finding. This trend aligns with South Korea’s aging population and highlights the growing burden of geriatric trauma [21,22]. Patients aged ≥ 65 years were more likely to sustain injuries from slips and falls, accounting for nearly 50% of trauma cases in this age group. These mechanisms were associated with higher ISS (median ISS nine [IQR 4–13]) and longer hospital stays (mean 15.0 ± 15.4 days). Although the proportion of patients with ISS > 15 was lower among those aged ≥ 65 years compared to those aged 20–64 years (19.9% vs. 22.0%), the case fatality rate was higher in the elderly population (6.1% vs. 3.2%). In addition, the proportion of super-elderly patients (aged ≥ 80 years) showed a sharper increase compared to younger elderly patients, along with a higher case fatality rate. It is well established that super-elderly patients have higher mortality and complication rates than younger elderly patients [23,24,25] and are also more likely to sustain severe injuries [26]. This suggests that the increase in overall mortality may be linked to the high fatality rates among elderly trauma patients and the rising proportion of super-elderly patients. Considering their limited physiological reserve and frequent comorbidities, elderly trauma patients may require a different approach than younger patients [27]. Developing specialized geriatric trauma care protocols, including consultations with geriatric medicine departments and age-appropriate rehabilitation services, is essential [28,29,30]. Second, the expansion of our trauma center may also contribute to this trend. The study results indicate that, while our trauma center treated an average of >3000 patients annually, the proportion of patients with ISS > 15 steadily increased, with a particularly notable rise in patients with ISS ≥ 25 (Table S1). Notably, the case fatality rate in this group was extremely high (26.4%), exceeding the overall average mortality rate (3.9%) (Table S6). A higher ISS generally reflects more complex or multiple injuries, thereby contributing to an elevated risk of mortality. The steady increase in the cumulative proportion of AIS three or higher injuries across anatomical regions may explain this trend. We surmise that, as the trauma center’s human and infrastructural resources matured, the increasing proportion of patients with higher injury severity may partly explain these outcomes [31].

However, the disproportionately high mortality among patients with ISS > 15 highlights the need for timely and effective management of severe trauma cases. Addressing the rising mortality requires enhanced trauma team education, improved trauma center infrastructure, and nationwide trauma prevention campaigns [32,33]. Enhancing prehospital care, expediting transport systems, and adopting advanced resuscitation protocols could play critical roles in mitigating this issue. Recent studies highlight the benefits of interventions, such as prehospital transfusion and tranexamic acid administration, in improving trauma outcomes [34,35,36]. However, structural limitations in South Korea’s trauma system, including legal restrictions on aggressive prehospital interventions, pose significant challenges. Rapid resuscitation during the early post-injury phase is crucial for improving survival in severe trauma cases [37,38]. Legislative and institutional reforms are necessary to enhance outcomes for these patients.

The main strength of this study lies in its detailed analysis of trends and outcomes over 10 years using a large trauma registry dataset. However, this study has several limitations. First, it is based on data from a single trauma center and may not fully represent South Korea’s national trauma care system, highlighting the need for further studies utilizing a national trauma registry. Second, while this study identified critical trends, additional investigations are required to elucidate the underlying factors driving these changes. Future research should explore the roles of socioeconomic factors, regional disparities, and advancements in prehospital and in-hospital trauma care on patient outcomes. Third, although we have consistently worked on establishing a regional trauma system and providing ongoing training for paramedics, we were unable to analyze the relationship between mortality and transport time to the Incheon Regional Trauma Center due to the limitations of our database. Given that one of the main goals of establishing trauma centers is to improve accessibility and quality of care, this could represent an important gap in our understanding. Lastly, this study did not analyze treatment outcomes. Further time-series analyses of complications, ICU-related outcomes, and long-term follow-up data could offer insights into the current state and future directions for improving treatment outcomes.

## 5. Conclusions

This study highlights a decade of experience at a regional trauma center, demonstrating evolving trends in patient demographics, injury mechanisms, and outcomes. The increasing proportion of elderly and severe trauma cases underscores the need for tailored protocols and continuous improvements in trauma care infrastructure to address the challenges of a changing patient population.

## Figures and Tables

**Table 1:** Demographic and Clinical Characteristics of Patients.

Characteristics	All Patients (n = 32,025)
Age, years, mean ± SD	52.8 ± 22.2
Male, n (%)	20,138 (62.9)
Mechanism, n (%)	
Traffic accident	9019 (29.9)
Fall	5923 (19.6)
Slip	8134 (26.9)
Struck by object	3378 (11.2)
Penetrating	2084 (6.9)
Others	1664 (5.5)
Season, n (%)	
Spring	7906 (24.7)
Summer	8168 (25.5)
Autumn	8590 (26.8)
Winter	7361 (23.0)
Time of week, n (%)	
Weekdays	22,523 (70.3)
Weekends	9502 (29.7)
Times of the day, n (%)	
Morning	12,078 (37.7)
Evening	13,747 (42.9)
Night	6200 (19.4)
Type of arrival to hospital, n (%)	
Direct	22,358 (70.2)
Transfer	9474 (29.8)
Initial triage, n (%)	
Trauma bay	10,711 (33.4)
ER	21,314 (66.6)
Initial Vital signs	
SBP, mmHg, mean ± SD	138.4 ± 30.4
HR, bpm, mean ± SD	86.8 ± 18.5
RR, breaths/min, mean ± SD	20.2 ± 3.4
GCS, median [IQR]	15 [15–15]
AIS ≥ 3, n (%)	
Head and Neck ≥ 3	5800 (18.1)
Face ≥ 3	117 (0.4)
Thorax ≥ 3	4581 (14.3)
Abdomen ≥ 3	1829 (5.7)
Extremity ≥ 3	5478 (17.1)
External ≥ 3	171 (0.5)
Injury Severity Parameter	
ISS, median [IQR]	9 [4–13]
1–8, n (%)	14,647 (48.5)
9–15, n (%)	9416 (31.1)
16–24, n (%)	3420 (11.3)
≥25, n (%)	2748 (9.1)
Hospital LOS, days mean ± SD	13.9 ± 16.8
Discharge disposition, n (%)	
Home	17,099 (53.6)
Transfer to another hospital	12,761 (40.0)
DOA (including death at ER)	774 (2.4)
Expired (after admission)	1256 (3.9)

Abbreviations: SD, standard deviation; ER, emergency room; SBP, systolic blood pressure; HR, heart rate; RR, respiratory rate; GCS, Glasgow Coma Scale; AIS, Abbreviated Injury Scale; IQR, interquartile range; ISS, Injury Severity Score; LOS, length of stay; DOA, death on arrival.

**Table 2:** Demographic and Clinical Characteristics of Patients with Trauma by Age Group.

Characteristics		Age Groups				*p* Value
	0–12	13–19	20–64	65–79	≥80	
Male, n (%)	1037 (64.7)	1024 (73.7)	13,369 (71.9)	3417 (52.3)	1291 (33.1)	<0.001
Mechanism, n (%)						<0.001
Traffic accident	378 (24.1)	585 (43.0)	5905 (33.1)	1672 (27.9)	479 (13.9)	
Fall	483 (30.8)	205 (15.1)	3613 (20.2)	1102 (18.4)	520 (15.1)	
Slip	300 (19.1)	174 (12.8)	3053 (17.1)	2418 (40.4)	2189 (63.6)	
Struck by object	228 (14.5)	173 (12.7)	2566 (14.3)	345 (5.8)	79 (2.2)	
Penetrating	78 (5.0)	149 (10.9)	1679 (9.4)	153 (2.6)	25 (0.7)	
Others	101 (6.4)	76 (5.6)	1040 (5.8)	297 (4.9)	153 (4.4)	
Injury Severity Parameter						
ISS, median [IQR]	4 [2–5]	4 [2–10]	6 [4–14]	9 [4–14]	9 [5–10]	<0.001
ISS > 15, n (%)	125 (7.9)	238 (17.5)	3936 (22.0)	1349 (22.6)	520 (15.2)	<0.001
Hospital LOS, days mean ± SD	5.3 ± 8.9	11.2 ± 15.2	14.3 ± 18.0	15.2 ± 16.3	14.6 ± 13.9	<0.001
Discharge disposition, n (%)						
Home	1274 (79.6)	946 (68.3)	10,030 (54.2)	3179 (48.9)	1670 (42.9)	<0.001

Abbreviations: IQR, interquartile range; ISS, Injury Severity Score; LOS, length of stay; SD, standard deviation.

**Table 3:** Mortality Rates by Age, Mechanism, and ISS.

Characteristics	Total (n)	Death (n)	Mortality Rate (%)
Age group			
0–12	1588	20	1.3
13–19	1352	32	2.4
20–64	18,076	584	3.2
65–79	6390	364	5.7
≥80	3830	255	6.7
Mechanism			
Traffic accident	8762	409	4.7
Fall	5522	304	5.5
Slip	8126	261	3.2
Struck by object	3345	35	1.0
Penetrating	2053	27	1.3
Others	1623	109	6.7
ISS			
1–8	14,503	79	0.5
9–15	9202	165	1.8
16–24	3202	222	6.9
≥25	2565	677	26.4

Abbreviations: ISS, Injury Severity Score.

## Data Availability

The data presented in this study are available on request from the corresponding author. The data are not publicly available due to privacy or ethical restrictions.

## Supplementary Materials

The following supporting information can be downloaded at https://www.mdpi.com/article/10.3390/healthcare13070773/s1, Table S1: Annual Number and Proportion of Patients by ISS Group; Table S2: Annual Number and Proportion of Patients with AIS ≥ 3 Injuries by Body Region; Table S3: Annual Number and Proportion of Patients by Injury Mechanism; Table S4: Annual Distribution of Patients with Trauma by Age Group (2014–2023); Table S5: Annual Survival and Mortality Rates by Age Group (2014–2023); Table S6: Annual Survival and Mortality Rates by ISS Group (2014–2023).

## Abbreviations

The following abbreviations are used in this manuscript:

ANOVA	analysis of variance
CPR	Cardiopulmonary resuscitation
ED	Emergency department
HLOS	Hospital length of stay
IQR	Interquartile range
IRB	Institutional review board
ISS	Injury Severity Score
KTDB	Korean Trauma Database
OECD	Organization for Economic Cooperation and Development
SPSS	Statistical Package for the Social Sciences
