# Supplementary material for: Epidemiology of Traumatic Injuries at a Single Regional Trauma Center in South Korea: Age-Specific and Temporal Trends (2014–2023)
Source: Healthcare (Basel). 2025 Mar 31;13(7):773. doi: 10.3390/healthcare13070773 (PMC11988991; doi:10.3390/healthcare13070773)
Supplement: Supplementary file 1 [file healthcare-13-00773-s001.zip › healthcare-3496089-supplementary.pdf]

Supplementary Tables

Supplement Table S1. Annual Number and Proportion of Patients by ISS Group

ISS group			Year										Total
			2014	2015	2016	2017	2018	2019	2020	2021	2022	2023	
0–8	Patients	No.	1,938	1,813	1,591	1,868	1,673	1,471	1,241	1,176	805	1,071	14,647
		%	59.3	57.0	52.9	54.7	52.1	48.2	42.6	41.9	32.3	37.1	48.5
9–15	Patients	No.	779	855	884	992	981	1003	1008	957	902	1055	9416
		%	23.8	26.9	29.4	29.1	30.6	32.8	34.6	34.1	36.2	36.5	31.1
16–24	Patients	No.	326	273	263	299	319	330	387	391	399	433	3420
		%	10.0	8.6	8.7	8.8	9.9	10.8	13.3	13.9	16.0	15.0	11.3
≥25	Patients	No.	226	237	268	254	238	251	277	282	386	329	2748
		%	6.9	7.5	8.9	7.4	7.4	8.2	9.5	10.0	15.5	11.4	9.1

Supplement Table S2. Annual Number and Proportion of Patients with AIS ≥3 Injuries by Body Region

Age group		Year										Total
		2014	2015	2016	2017	2018	2019	2020	2021	2022	2023	
Head&Neck AIS≥3	No.	524	530	597	582	609	577	569	567	633	612	5,800
	%	16.0	16.7	19.8	17.0	18.9	18.5	17.5	17.5	21.6	18.2	18.1
Face AIS≥3	No.	11	10	16	7	5	7	15	15	15	16	117
	%	0.3	0.3	0.5	0.2	0.2	0.2	0.5	0.5	0.5	0.5	0.4
Thorax AIS≥3	No.	325	361	379	420	431	438	500	527	588	612	4,581
	%	9.9	11.4	12.6	12.3	13.4	14.1	15.3	16.2	20.1	18.2	14.3
Abdomen AIS≥3	No.	139	152	181	162	146	141	222	206	238	242	1,829
	%	4.3	4.8	6.0	4.7	4.5	4.5	6.8	6.3	8.1	7.2	5.7
Extremity AIS≥3	No.	407	423	482	546	518	628	629	597	576	672	5,478
	%	12.5	13.3	16.0	16.0	16.1	20.2	19.6	18.4	19.7	19.9	17.1
External AIS≥3	No.	15	13	21	21	21	16	16	17	19	12	171
	%	0.5	0.4	0.7	0.6	0.7	0.5	0.5	0.5	0.6	0.4	0.5

Supplement Table S3. Annual Number and Proportion of Patients by Injury Mechanism

Cause of trauma	2014 *n* (%)	2015 *n* (%)	2016 *n* (%)	2017 *n* (%)	2018 *n* (%)	2019 *n* (%)	2020 *n* (%)	2021 *n* (%)	2022 *n* (%)	2023 *n* (%)
Traffic accident	1,004 (30.7)	1,027 (32.3)	905 (30.0)	1,028 (30.0)	997 (31.0)	1,005 (32.7)	793 (27.3)	739 (26.5)	714 (28.9)	807 (28.2)
Fall	501 (15.3)	679 (21.4)	588 (19.5)	645 (18.9)	654 (20.3)	578 (18.8)	530 (18.3)	580 (20.8)	566 (22.9)	602 (21.0)
Slip	878 (26.9)	583 (18.3)	711 (23.6)	777 (22.7)	771 (23.9)	851 (27.7)	953 (32.9)	905 (32.4)	731 (29.6)	974 (34.0)
Struck by object	402 (12.3)	388 (12.2)	402 (13.3)	500 (14.6)	400 (12.4)	308 (10.0)	320 (11.0)	249 (8.9)	205 (8.3)	204 (7.1)
Penetrating	243 (7.4)	269 (8.5)	218 (7.2)	288 (8.4)	253 (7.9)	189 (6.2)	193 (6.7)	176 (6.3)	132 (5.3)	123 (4.3)
Others	241 (7.4)	234 (7.4)	189 (6.3)	183 (5.3)	145 (4.5)	139 (4.5)	112 (3.9)	144 (5.2)	121 (4.9)	156 (5.4)

Supplement Table S4. Annual Distribution of Patients with Trauma by Age Group (2014–2023)

Age group		Year										Total
		2014	2015	2016	2017	2018	2019	2020	2021	2022	2023	
0–12	No.	237	227	225	187	178	150	112	127	69	91	1,603
	%	7.2	7.1	7.5	5.5	5.5	4.8	3.4	3.9	2.4	2.7	5.0
13-19	No.	209	198	130	170	163	111	103	79	114	112	1,389
	%	6.4	6.2	4.3	5.0	5.1	3.6	3.2	2.4	3.9	3.3	4.3
20–64	No.	2,062	2,001	1,869	2,137	1,933	1,841	1,821	1,740	1,550	1,645	18,599
	%	63.1	62.9	62.0	62.5	60.0	59.1	55.9	53.6	52.9	48.8	58.1
65–79	No.	549	537	528	624	638	611	734	756	662	891	6,530
	%	16.8	16.9	17.5	18.2	19.8	19.6	22.5	23.3	22.6	26.4	20.4
≥80	No.	212	217	261	303	308	402	489	545	535	632	3,904
	%	6.5	6.8	8.7	8.9	9.6	12.9	15.0	16.8	18.3	18.7	12.2
Overall	No.	3,269	3,180	3,013	3,421	3,220	3,115	3,259	3,247	2,930	3,371	32,025
	%	100.0	100.0	100.0	100.0	100.0	100.0	100.0	100.0	100.0	100.0	100.0

Supplement Table S5. Annual Survival and Mortality Rates by Age Group (2014–2023)

Age group			Year										Total
			2014	2015	2016	2017	2018	2019	2020	2021	2022	2023	
0–12	Survival	No.	233	227	223	184	173	143	107	123	68	87	1,568
		%	98.7	100	99.1	99.5	97.7	97.9	98.2	96.9	100	98.9	98.7
	Expired	No.	3	0	2	1	4	3	2	4	0	1	20
		%	1.3	0.0	0.9	0.5	2.3	2.1	1.8	3.1	0.0	1.1	1.3
13-19	Survival	No.	202	195	128	167	152	102	93	75	102	104	1,320
		%	98.5	99.0	98.5	98.8	94.4	97.1	93.9	96.2	100	98.1	97.6
	Expired	No.	3	2	2	2	9	3	6	3	0	2	32
		%	1.5	1.0	1.5	1.2	5.6	2.9	6.1	3.8	0.0	1.9	2.4
20–64	Survival	No.	1,980	1,901	1,791	2,046	1,802	1,730	1,715	1,623	1,393	1,511	17,492
		%	97.6	97.4	96.8	98.4	94.8	96.8	97.6	96.8	95.0	95.7	96.8
	Expired	No.	49	50	59	34	98	57	43	53	73	68	584
		%	2.4	2.6	3.2	1.6	5.2	3.2	2.4	3.2	5.0	4.3	3.2
65–79	Survival	No.	517	502	502	592	582	561	689	682	590	809	6,026
		%	95.4	96.4	95.8	96.7	93.3	93.8	96.0	92.5	92.0	92.7	94.3
	Expired	No.	25	19	22	20	42	37	29	55	51	64	364
		%	4.6	3.6	4.2	3.3	6.7	6.2	4.0	7.5	8.0	7.3	5.7
≥80	Survival	No.	197	192	243	283	284	373	443	495	481	584	3,575
		%	94.3	91.4	93.8	95.0	93.4	94.7	91.9	93.2	92.1	94.0	93.3
	Expired	No.	12	18	16	15	20	21	39	36	41	37	255
		%	5.7	8.6	6.2	5.0	6.6	5.3	8.1	6.8	7.9	6.0	6.7
Overall	Survival	No.	3,129	3,017	2,887	3,272	2,993	2,909	3,047	2,998	2,634	3,095	29,981
		%	97.1	97.1	96.6	97.8	94.5	96.0	96.2	95.2	94.1	94.7	96.0
	Expired	No.	92	89	101	72	173	121	119	151	165	172	1,255
		%	2.9	2.9	3.4	2.2	5.5	4.0	3.8	4.8	5.9	5.3	3.9

Supplement Table S6. Annual Survival and Mortality Rates by ISS Group (2014–2023)

ISS group			Year										Total
			2014	2015	2016	2017	2018	2019	2020	2021	2022	2023	
0–8	Survival	No.	1,930	1,797	1,577	1,842	1,623	1,439	1,217	1,161	786	1,052	14,424
		%	99.6	99.8	99.4	99.7	98.4	99.7	99.6	99.4	99.4	99.5	99.5
	Expired	No.	7	4	10	5	27	4	5	7	5	5	79
		%	0.4	0.2	0.6	0.3	1.6	0.3	0.4	0.6	0.6	0.5	0.5
9–15	Survival	No.	763	820	869	961	934	964	969	902	843	1,012	9,037
		%	99.2	98.7	98.6	99.2	96.2	98.2	98.3	97.9	97.9	98.2	98.2
	Expired	No.	6	11	12	8	37	18	17	19	18	19	165
		%	0.8	1.3	1.4	0.8	3.8	1.8	1.7	2.1	2.1	1.8	1.8
16–24	Survival	No.	284	241	242	269	269	284	339	339	346	367	2,980
		%	90.4	93.1	94.9	94.4	87.1	91.6	95.2	94.2	95.3	93.9	93.1
	Expired	No.	30	18	13	16	40	26	17	21	17	24	222
		%	9.6	6.9	5.1	5.6	12.9	8.4	4.8	5.8	4.7	6.1	6.9
≥25	Survival	No.	152	157	197	199	164	173	193	187	250	216	1,888
		%	75.6	73.7	76.1	82.2	70.7	72.1	75.1	71.4	71.2	70.1	73.6
	Expired	No.	49	56	62	43	68	67	64	75	101	92	677
		%	24.4	26.3	23.9	17.8	29.3	27.9	24.9	28.6	28.8	29.9	26.4
